# Draft Genome of the Asian Buffalo Leech *Hirudinaria manillensis*

**DOI:** 10.3389/fgene.2019.01321

**Published:** 2020-01-16

**Authors:** De-Long Guan, Jie Yang, Ying-Kui Liu, Yuan Li, Da Mi, Li-Bin Ma, Zhe-Zhi Wang, Sheng-Quan Xu, Qiang Qiu

**Affiliations:** ^1^College of Life Sciences, Shaanxi Normal University, Xi'an, China; ^2^Center for Ecological and Environmental Sciences, Northwestern Polytechnical University, Xi'an, China; ^3^College of Biomedical Sciences & Department of Biological Sciences, Xuzhou Medical University, Xuzhou, China; ^4^Nextomics Biosciences Institute, Wuhan, China

**Keywords:** Asian buffalo leech, *Hirudinaria manillensis*, sequencing, genome assembly and annotation, Hirudin/Antistasin gene family

## Abstract

The Asian Buffalo leech, *Hirudinaria manillensis*, is an aquatic sanguivorous species distributed widely in Southeast Asia. *H. manillensis* has long been used clinically for bloodletting and other medical purposes. Recent studies have focused on artificial culturing, strain optimization, and the identification and development new drugs based on the anticoagulant effects of *H. manillensis* bites; however, data regarding its genome remain unclear. This study aimed to determine the genome sequence of an adult Asian Buffalo leech. We generated a draft assembly of 151.8 Mb and a N50 scaffold of 2.28 Mb. Predictions indicated that the assembled genome contained 21,005 protein-coding genes. Up to 17,865 genes were annotated in multiple databases including Gene Ontology. Sixteen anticoagulant proteins with a Hirudin or Antistasin domain were identified. This study is the first to report the whole-genome sequence of the Asian Buffalo leech, an important sanguivorous leech of clinical significance. The quality of the assembly is comparable to those of other annelids. These data will help further the current understanding of the biological mechanisms and genetic characteristics of leeches and serve as a valuable resource for future studies.

## Introduction

**Figure 1:**
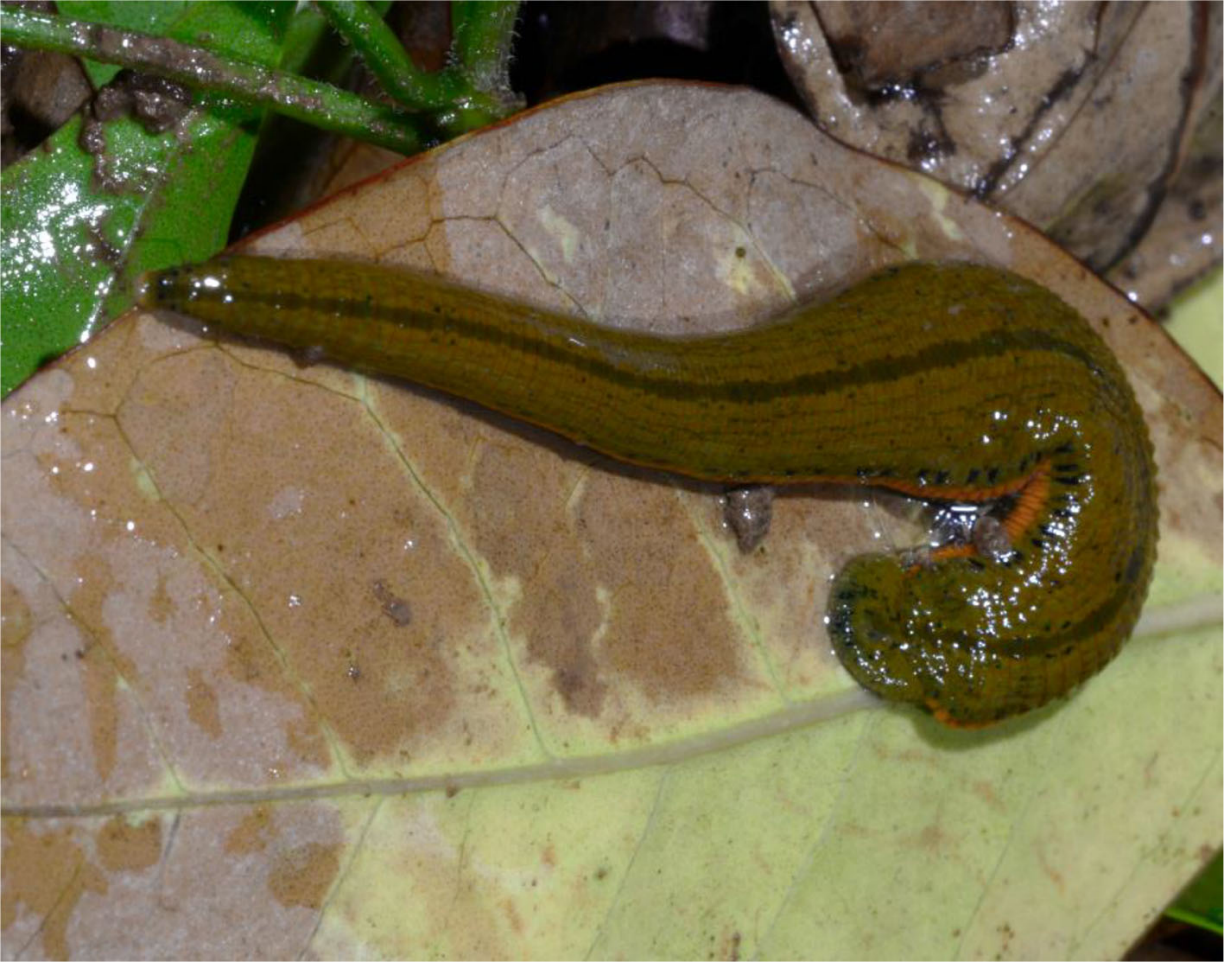
The adult Asian Buffalo leech, *Hirudinaria manillensis* (image copy-right retrieved from Li-Bin Ma).

The Asian Buffalo leech, *Hirudinaria manillensis* (NCBI taxonomy ID: 1348078) (**Figure 1**) is a member of family Hirudinidae, order Arhynchobdellida, and phylum Annelida. *H. manillensis* is a type of hermaphrodite segmented worm endemic to southeast Asia (Liu et al., 2015; Oliver and DeLoughery, 2019). The leech is widely distributed in the Philippines, Vietnam, Malaysia, and several provinces of China including the Guangxi, Hunan, and Fujian provinces (Sket and Trontelj, 2007; Liu et al., 2015).

*H. manillensis* displays various fascinating behavioral and physiological characteristics that are of interest to evolutionary, biochemical, and pharmaceutical studies. *H. manillensis* differs from oligochaetes, which typically have 2 suckers at each end of the body, by having independent internal and external body segmentations and a robust muscle coelom; these properties make *H. manillensis* a good model for studying the evolution of the annelid body plan (Muller et al., 1981; Apakupakul et al., 1999; Struck et al., 2007; Teut and Warning, 2008; Oliver and DeLoughery, 2019). Moreover, *H. manillensis* is a sanguivorous species, which is an important behavioral aspect in leeches with clinical significance (Chiang et al., 2000; Thompson, 2010; Kvist et al., 2011; Gãdekmerdan et al., 2011). The emergence of sanguivorous behavior and efficient anti-coagulation secretions provide important insights into specific adaptions of medicinal leeches (de Koning et al., 2000; Striepen et al., 2004). The jaw of *H. manillensis* is tripartite and filled with hundreds of tiny, sharp teeth for attaching to mammalian hosts such as cattle, horses, and humans. Studies have described the anticoagulant effects of *H. manillensis* bites, as sites bleed copiously even hours after leech detachment (Elliott and Sawyer, 1987; Teut and Warning, 2008). This anticoagulant effect is considered the most important feature of *H. manillensis* and has facilitated its application for clinical bloodletting and other clinical applications (Sawyer, 1981; Abdelgabar and Bhowmick, 2003). The baked whole body of *H. manillensis* is an important ingredient in Chinese traditional medicine and is used to promote blood circulation and relieve gore (Chiang et al., 2000). Recent increases in the incidence of blood clots and stroke have led to numerous studies on the utility and market potential of *H. manillensis* products.

An increasing number of studies have focused on artificial culturing, strain optimization, and the identification and development of new drugs based on this species; nonetheless, genome and genetic data are unavailable for this species. Thus far, the genome of only one non-blood sucking leech, *Helobdella robusta*, has been characterized and applied for studies on its bilateral symmetry (Simakov et al., 2013). Even at the phylum level, the genome sequences of only three additional species, *Capitella teleta*, *Hydroides elegans*, and *Eisenia fetida*, are available (Veenstra, 2011; Zwarycz et al., 2015), and low-coverage genomic data are available for two lineages of *Amynthas cortices* for microsatellite analysis (Cunha et al., 2017). Accordingly, the whole-genome characterization of *H. manillensis* would be of great value with respect to its genetic foundations and mechanisms; therefore, this study aimed to determine the genome sequence of an adult Asian Buffalo leech, *Hirudinaria manillensis*.

## Methods

### Sample Collection and Sequencing

Genomic DNA was extracted from muscle tissue dissected from the body of a single adult Asian Buffalo leech captured in a rice field in HeChi city, Guangxi Province. This species is an invertebrate and there are no restrictions on capturing adult leeches. DNA extraction was performed using a QIAamp DNA extraction kit (Qiagen, Hilden, Germany) in accordance with the manufacturer specifications. In total, 20 μg DNA was retrieved and used for genome sequencing.

A short-insert library was sequenced using the Hiseq 4000 platform (Illumina, San Diego, CA, USA) to generate an initial survey in accordance with the standard protocol. In detail, a part of the DNA sample was randomly fragmented using an ultrasonic fragmentation apparatus. The library was prepared in accordance with the procedure of end repair, adding adaptor, purification, and PCR amplification. In total, 12.3 Gb of raw reads were obtained. Through prior whole-genome sequencing, a 20 k SMRTcell library was constructed using the standard protocol of Pacbio (Pacific Biosciences, San Diego, CA, USA). Sequencing was performed in two SMRT cells on the Pacbio sequel platform. Brieﬂy, the DNA sample was initially trimmed using Diagenode Megaruptor2 (Luxembourg, Belgium), and universal hairpin adapters were ligated onto double-stranded DNA fragments. Adapter dimers were eliminated using Pacific Biosciences' (PacBio's) MagBead kit. Thereafter, the failed ligation DNA fragments were eliminated using exonucleases. After exonuclease and AMPure PB purification, sequencing primers were annealed to the SMRTbell templates to facilitate the binding of the sequence polymerase. In total, approximately 15.1 Gb (~100X) of long raw reads were generated. Statistics for genome sequencing are listed in **Table S1** (**Supplementary Data Sheets 1**: **Table S1**).

### Quality Control of Sequenced Reads

For short reads, the Trimmomatic java package (Trimmomatic, RRID: SCR 011848, Version 0.38) (Bolger et al., 2014) was used to eliminate adapter-contaminated reads (defined as matches with the adapter sequence library >10 bp), low-quality or empty reads (defined as reads with >5% undetermined bases or a Phred quality score <30). After processing, we obtained 12.2 Gb of clean data. Regarding the Pacbio sequel data, we obtained approximately 14.9 Gb of clean data after eliminating adapter-contaminated reads and low-quality reads <500 bp, using Proovread (Version 2.12), an error correction package included in the CANU (CANU, RRID: SCR_015880, Version 2.12) (Koren et al., 2017). Overall, we retained 98.6% of the raw Pacbio sequel data including 1,505,497 reads.

### Nuclear Genome Assembly

We used CANU (CANU, RRID: SCR_015880, Version 2.12) (Koren et al., 2017), LoRDEC (LoRDEC, RRID: SCR_015814, Version 0.3) (Salmela and Rivals, 2014), and MECAT (Xiao et al., 2017) packages (https://github.com/xiaochuanle/MECAT) for sequence calibration and genome assembly. This assembly process references a previous successful assembly attempt using MECAT software (Liu et al., 2019). In brief, we first used CANU to self-correct the clean Pacbio long-reads to rectify random sequencing errors (Koren et al., 2017). Thereafter, all reads were readjusted using the short reads survey data, with LoRDEC (Salmela and Rivals, 2014) to exclude the possibility of faulty bases. Finally, calibrated reads were deposited into MECAT for complete assembly.

### RNA Extraction, Transcriptome Sequencing, and Processing of Reads

Muscle tissues from six additional individuals of *H. manillensis* were obtained for transcriptome sequencing to improve the findings of genome assembly and to determine gene expression levels (Denton et al., 2014). These individuals share same breeding line with the individual used for DNA extraction and was captured from the same natural population, that is the rice field in HeChi city, Guangxi Province. To determine the differentially expressed genes associated with feeding, these individuals were segregated into two groups and have undergone different treatments through starving and blood-feeding.

RNA extraction was performed using a RNeasy Mini Kit (Qiagen, Hilden, Germany) in accordance with the manufacturer's instructions. Two cDNA libraries with inserted sequence sizes of 150 kb were constructed for sequencing using the Hiseq 4000 platform (Illumina, San Diego, CA, USA) for transcriptome sequencing. In brief, approximately 3 μg of total RNA for each sample was used to prepare RNA-seq libraries. The mRNA was enriched using poly‐T oligo‐attached magnetic beads from the total RNA and subsequently randomly fragmented in fragmentation buffer (Biomarker, Beijing, China). Thereafter, the fragments were used as the template for cDNA synthesis using random hexamers. Retrieved double‐strand cDNA fusion was achieved and purified using AMPure XP beads (Beckman Coulter, California, USA). Finally, the final cDNA libraries with the reads of preferentially 150–200 bp were selected with the AMPure XP system. In total, raw reads of 42.81 Gb were obtained.

Using the RNA-seq data, a referenced genome‐guided mapping against the assembly of *H. manillensis* was performed for each of the paired‐end samples, using Hisat2 (HISAT2, RRID: SCR_015530) (Kim et al., 2015). The stringtie software (StringTie, RRID: SCR_016323, Version 2.0) (Pertea et al., 2015) was then used to determine the FPKM values of all genes to perform differential expression analysis of the two conditions (fasting and feeding).

### Genome Assembly Assessment

All data that retrieved from Illumina sequencing were aligned to the assembled genome. The generated bam file was handled using Samtools (Samtools, RRID: SCR_002105) (Li et al., 2009) and finally used to check and adjust genome accuracy, using Pilon (Pilon, RRID: SCR_014731) (Walker et al., 2014). Furthermore, Benchmarking Universal Single-Copy Orthologs (BUSCO, RRID: SCR 015008) (Simao et al., 2015) was used to check the completeness of the genome assembly based on metazoan core genes. BUSCO was used with the metazoan orthologous gene set, and the complete, fragment, and lost genes were determined in the assembly.

### Determination of Repetitive Elements

We determined repetitive sequences using RepeatMasker (RepeatMasker, RRID: SCR_012954) and RepeatModeler (RRID: SCR_015027) pipeline (Huda and Jordan, 2009). RepeatModeler was used to determine a consensus sequence for each repeat family. Results from RepeatModeler and Annelida repeat sequence database were combined. This combined database was used to annotate and mask repetitive sequences in the *H. manillensis* genome. The results are presented in **Table S2** (**Supplementary Data Sheet 2**: **Table S2**).

### Gene Prediction

We combined *Ab initio* and homology-based prediction methods to construct consensus gene models. We used PASA (PASA, RRID: SCR_014656) (Haas et al., 2003), Genscan (GENSCAN, RRID: SCR_012902) (Burge and Karlin, 1997), Augustus (Augustus: Gene Prediction, RRID: SCR 008417) (Stanke et al., 2006), GlimmerHMM (GlimmerHMM, RRID: SCR_002654) (Majoros et al., 2004), GeneID (GeneID, SCR_002473) (Alioto et al., 2018), and SNAP (Korf, 2004) to search for gene models. Using the homology-based method, protein sequences of *Caenorhabditis elegans* (nematode, GCA_000002985.3), *Capitella teleta* (Marine worm, GCA_000328365.1), and *Helobdella robusta* (freshwater leech, GCA_000326865.1) were downloaded from the NCBI GenBank database (GenBank, RRID: SCR_002760) (Benson et al., 2014) and implemented for library construction. The data were deposited into GeMoMa (Keilwagen et al., 2016), which is a jar package integrated into an analysis pipeline. Finally, gene models from the *Ab initio* and homology-based prediction methods were combined into a non-redundant gene set of 21,005 genes, using EVidenceModeler (EVidenceModeler (EVM), RRID: SCR_014659) software (**Supplementary Data Sheet 2**: gene_finding.gff) (Haas et al., 2008).

### Gene Function Annotation

For functional annotation, the Pfam database (PFAM, RRID: SCR_004726) (Punta et al., 2011) was used to annotate proteins, using HMMER (HMMER, RRID: SCR_005305) (Finn et al., 2011). Gene Ontology (GO; GO, RRID: SCR 002811) (Sherlock, 2009) terms were obtained using blast2go (Blast2GO, RRID: SCR_005828) (Conesa et al., 2005). Metabolic functions of these genes were assigned using the Kyoto Encyclopedia of Genes and Genomes (KEGG, RRID: SCR 012773) database (Kanehisa and Goto, 2000). The motifs and domains of genes were determined using InterProScan (InterProScan, RRID: SCR 005829) (Zdobnov and Apweiler, 2001) against the InterProscan protein signature databases including HAMAP (HAMAP, RRID: SCR_007701) (Lima et al., 2009), PRINTS (PRINTS, RRID: SCR 003412) (Attwood et al., 2003), Pfam (Pfam, RRID: SCR 004726) (Punta et al., 2011), SMART (SMART, RRID: SCR 005026) (Letunic et al., 2011), PANTHER (PANTHER, RRID: SCR 004869) (Mi et al., 2010), and ProDom (ProDom, RRID: SCR 006969) (Servant et al., 2002). All annotations are shown in **Supplementary Data Sheet 1**.

### Data Analysis and Visualization

Synteny analysis was conducted using MCscanX (Wang et al., 2012). GO and KEGG enrichment analyses were conducted using KOBAS 3.0 (KOBAS, RRID: SCR_006350). Tables and figures were visualized using Geneious, TBtools (Chen et al., 2018) and MS Excel.

## Results and Discussion

### Genome Sequencing and Assembly

A high-quality draft genome of *H. manillensis* was obtained. The resulting assembly included 467 scaffolds with a total length of 151.8 Mb. A comparison between the assembled genome size with the kmer-based estimated size is a common measurement to reveal missing sequences. Using the obtained short reads, the whole genome length was approximately 160.5 Mb, suggesting that our assembly is approximately 94.5% complete with few missing sequences.

The length of scaffolds is highly variable. The present assembly displayed a scaffold N50 of 2.28 Mb and an overall GC content of 35.98%. The longest scaffold was of 5.92 Mb and the median scaffold length was 17,504 bp. These parameters are comparable with the genome assemblies of other annelids and the most outstanding advantage for the present assembly is the marked reduction in the number of scaffolds (**Table 1**). The enhanced connectivity of scaffolds is reflected in the average length, which is 324,782 bp. Compared with that of *H. robusta*, the present scaffold is approximately 2.74-fold longer.

**Table 1:** Comparison of the genome assembly and number of genes with published genomes of other annelids.

	Genome Len (bp)	Seq Num	GC content (%)	Minimum Len (bp)	Max Len (bp)	Mean Len (bp)	Median Len (bp)	N50 (bp)
***Hirudinaria manillensis***	151,673,145	467	0.36	2,693	5,920,317	324,782	17,504	2,277,518
***Helobdella robusta* (GCA_000326865.1)**	235,376,169	1,991	0.33	1,000	13,640,604	118,220	3,236	3,060,193
***Capitella teleta* (GCA_000328365.1)**	333,283,208	20,803	0.40	1,000	1,620,044	16,021	2,479	188,402
***Eisenia fetida* (GCA_003999395.1)**	1,471,976,452	399,003	0.40	500	91,445	3,689	764	9,314
***Eisenia fetida* (GCA_900000155.1)**	1,052,631,503	1,659,527	0.41	100	58,500	634	182	1,852
***Hydroides elegans* (GCA_001703475.1)**	1,026,046,400	188,407	0.35	300	244,066	5,446	1,225	17,329

Len, length; seq num, sequence number.

Thereafter, we evaluated the completeness of the *H. manillensis* assembly by searching for 978 metazoa-conserved BUSCOs (Simao et al., 2015). All repetitive elements were pre-masked to prevent unnecessary intervention. Consequently, 897 (91.7%) complete BUSCOs and 20 (2.0%) fragmented BUSCOs were identified, while only 61 (6.3%) BUSCOs were missing from the assembly. Based on a previous estimation of assembly integrity, these numbers also suggested that the present assembly is approximately 93.7% (complete and fragmented BUSCOs) complete and has high quality.

**Figure 2:**
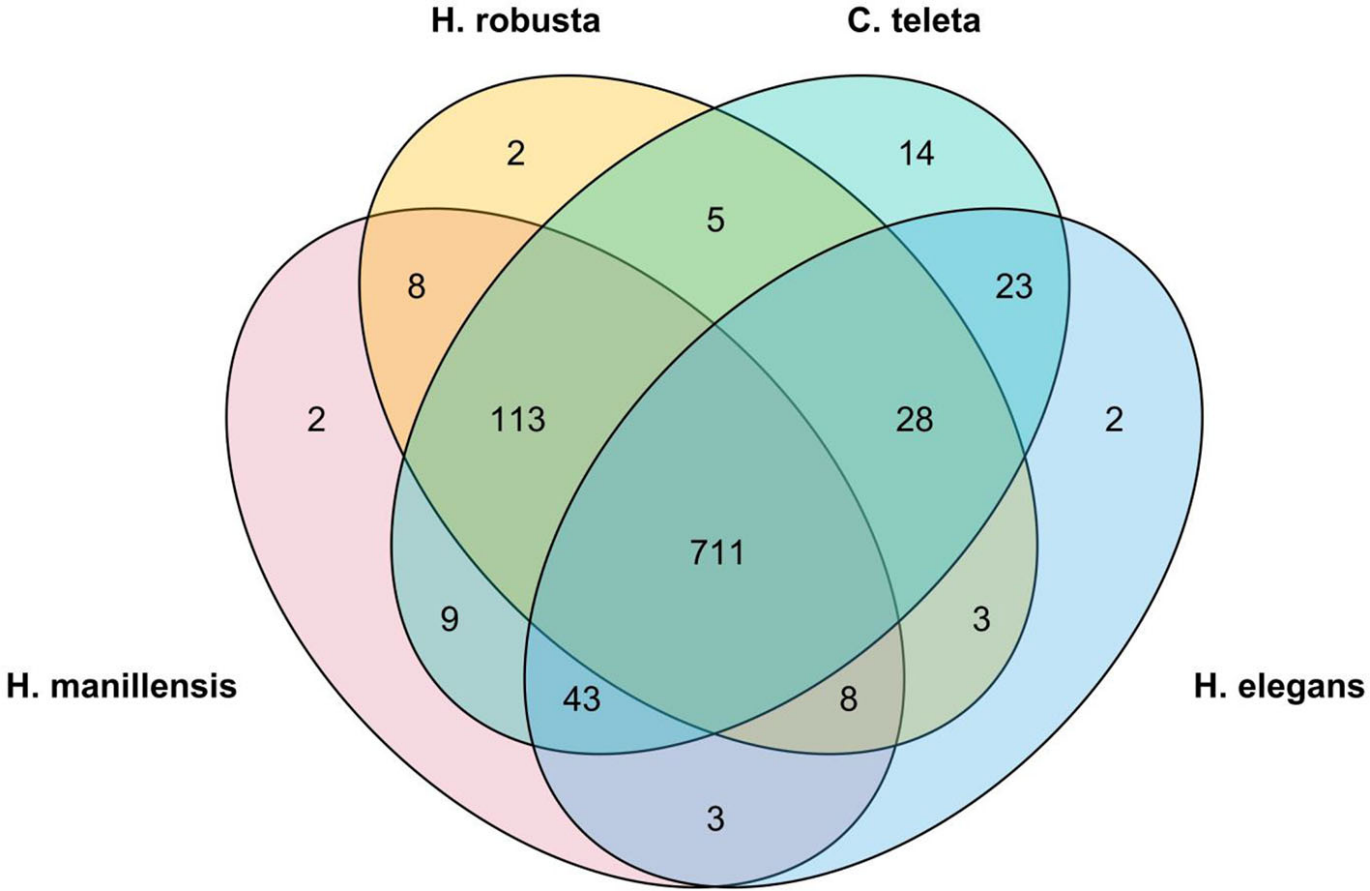
Venn plot of the common identified BUSCOs among all four published annelids' genome assemblies.

Furthermore, we performed BUSCO analysis for other published annelid genomes, using the same parameters and compared the completeness of each genome assembly (**Figure 2**). Both assemblies of *Eisenia fetida* did not yield long scaffolds and the maximum length was only 91,445 and 58,500 bp; therefore, they were excluded from further comparative genomic analysis. The quality of the *H. manilensis* assembly was comparable with that of others, and most of the determined BUSCOs (711) were shared among these four genomes. The number of identified complete BUSCOs was greater in the present assembly than in that of *H. robusta* (91.7 to 89.7%).

### Determination of Repetitive Elements

RepeatMasker and RepeatModeler pipeline analyses revealed that the *H. manillensis* assembly comprises 19.52% (29.6 Mb) of repetitive elements (**Table S2**). This proportion of repetitive elements was greater than that of *H. robusta* (17.33%). Hence, some repetitive elements resembled protein-coding sequences; they were subsequently replaced use N to facilitate the accuracy of gene identification. In addition, by combining the genome size analysis of these two species, it was not difficult to determine that *H. robusta* has a larger genome size, but a smaller proportion of repeat sequences, contrary to previous reports regarding the structure and composition of the genome. Therefore, we speculate that the underlying reason may be the difference in sequencing technology when obtaining the genome assembly. Previously, genome acquisition of *H. robusta* was carried out through next-generation short-read-length sequencing technology. Because the length of a single read is limited to several hundred base pairs (usually 150 or 250 bp), the repeated fragments on the genome usually cannot be correctly interpreted, resulting in the deletion and reduction of repeat sequences in the assembly. However, with third-generation technology, its sequencing reads can approach a read length of more than 20 kb, thus greatly enhancing its ability to span high complex repeating fragments of the genome and significantly increasing the number of identified repeat sequences and improve the quality and accuracy of the genome assembly.

### Gene Identification and Annotation

Using the masked assembly, combining the *Ab initio* and homology-based prediction method, we finally identified 21,005 protein coding genes in *H. manillensis* genome, with high confidence. The total length of all genes was 62.23 Mb, representing 41.03% of the genome. The average length of genes and CDSs are 2,963.1 bp and 1,293.3 bp, respectively. Mean protein sequence length is 431.1 aa (amino acids). A BUSCO assessment was conducted to evaluate the completeness of these determined genes, revealing 90.1% complete BUSCOs. Almost all BUSCOs predicted from the genome assembly were present in the identified genes, and these gene models had high integrity.

**Figure 3:**
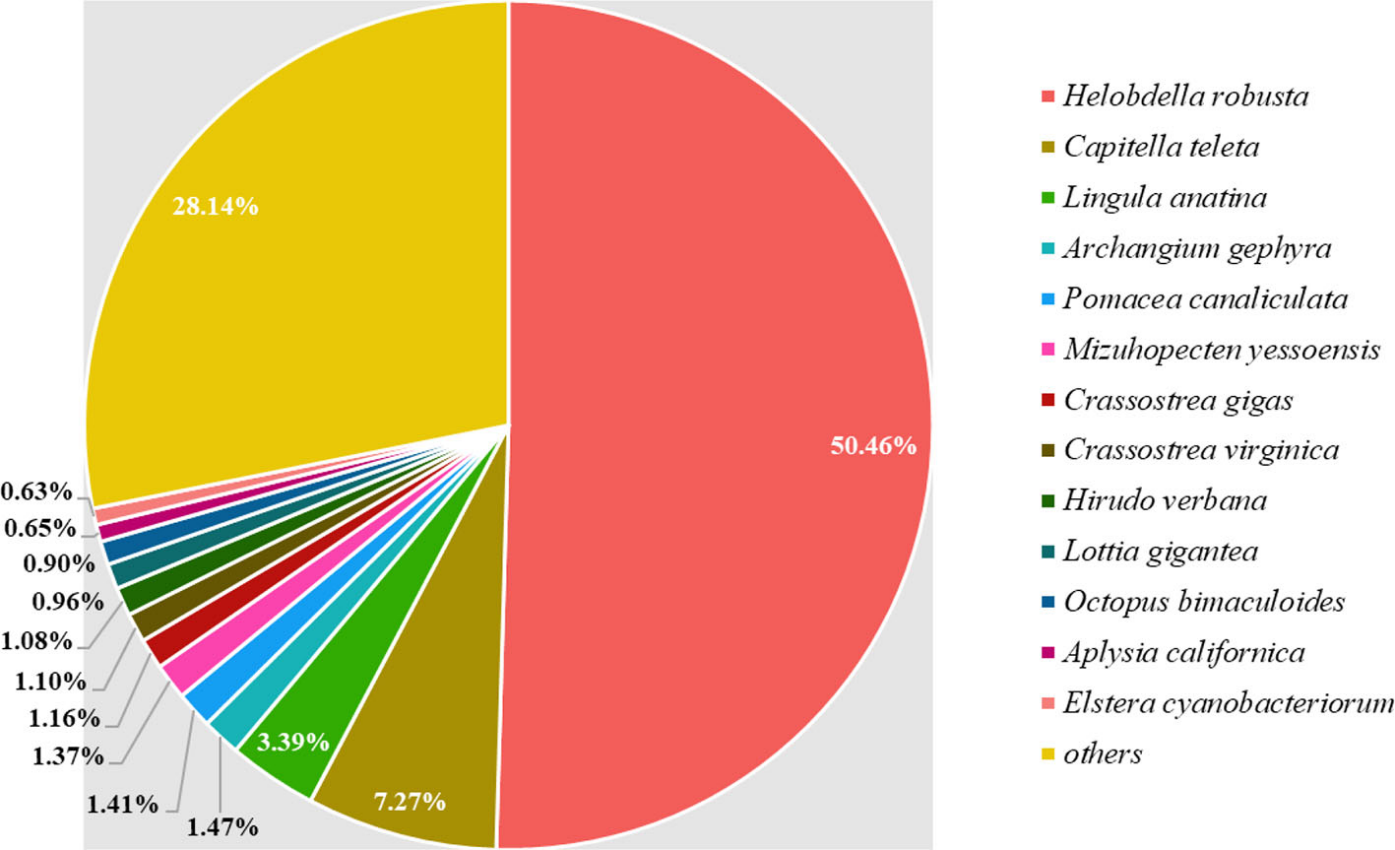
Distribution of NR annotations retrieved from different source species. Each of the species is denoted by a different color. The “others” section in the figure represents species occupying neglectable proportions.

Regarding gene annotation, 17,865 genes showed high-confidence matches (E-value ≤ 1e-5) in the Pfam (14,287 genes), GO (16,050 genes), KEGG (6,435 genes), interproscan (16,588 genes), and NCBI's non-redundant (NR) protein databases (16, 945), accounting for 85.05% of total number of genes. The source species of annotations retrieved from the NR were traced (**Figure 3**), and 50.46% were known sequences from *H. robusta*, which was the best match. As species of leeches, they share similar genetic backgrounds. Furthermore, *C. teleta* and *Lingula anatina* contribute to 7.27 and 3.39% of NR annotations and are distant relatives of leeches from the superorder Lophotrochozoa.

**Figure 4:**
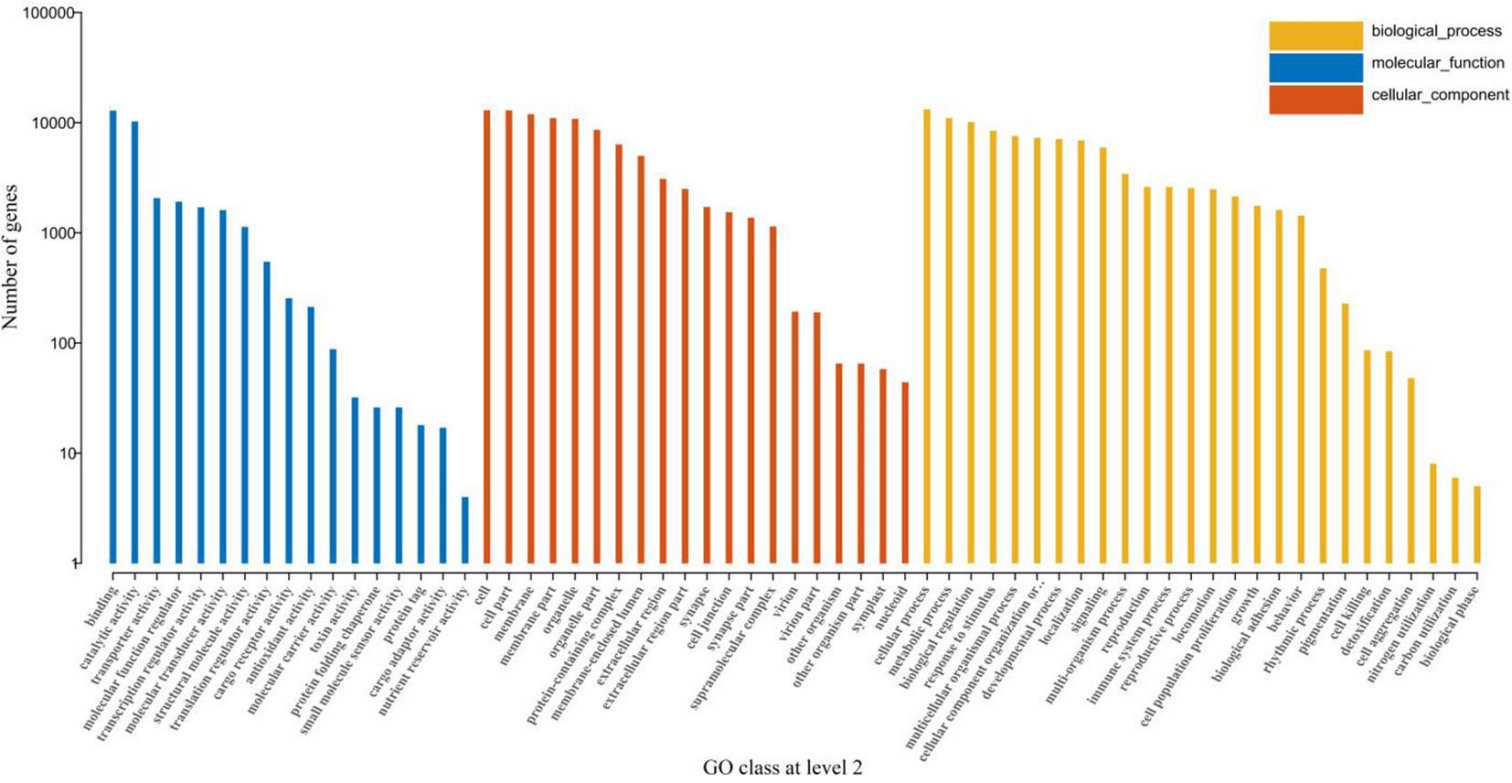
GO classification at level 2. The lateral-axis shows the GO terms, while the vertical-axis shows the number of genes accounted for in each term. The blue, red, and orange bars represent different GO classifications of the molecular function, cellular component, and biological process, respectively.

GO is a standardized gene functional classification system, which comprehensively describes the properties of genes and gene products in organisms. We classified all the retrieved GO terms and found that biological processes related to vital metabolic pathways were most enriched in terms of cellular processes, biological regulation, response to stimuli, *etc*. (**Figure 4**).

### Intraspecific/Interspecific Synteny

**Figure 5:**
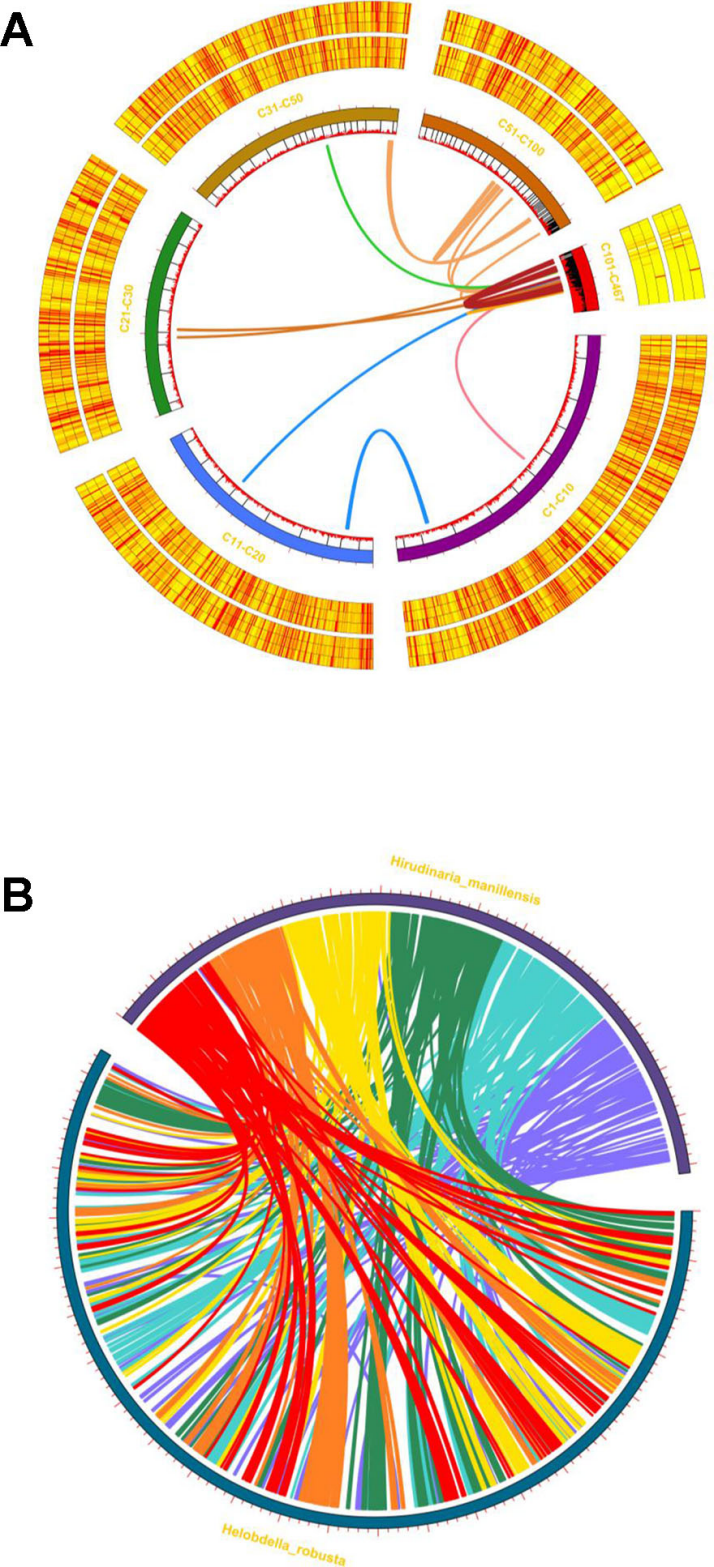
Intraspecific/interspecific MCscanX synteny analyses. **(A)** Whole-genome syntenic mapping of *H. manillensis* genes to itself. The innermost lines represent synteny linkage; the innermost circle represents the gene distribution, and the tagged circles (C1-C10, C11-C20, C21-C30, C31-C50, C51-C100,C101-C467) represent different merged contigs; the outsider heatmap circles represent the expression level (six samples, before and after feeding, from inner to outside). **(B)** Circular view of the synteny between the *H. manillensis* genes and the *H. robusta* genes.

Intraspecific/interspecific synteny analysis was performed using MCscanX (**Figure 5A, B**), revealing 246 syntenic gene blocks (in 123 pairs), representing 1.17% of the total protein coding sequences (**Figure 5A**). The *H. manillensis* genome has few intraspecific collinear genes, indicating that genome-wide gene duplication has a limited effect during long-term evolution in this species. Moreover, the interspecific gene synteny linkage between the genome of *H. manillensis* and *H. robusta* revealed 9,383 interspecific syntenic gene blocks (21.12% of all genes) of 4,724 pairs among them (**Figure 5B**). These two species share genome-wide syntenic linkage, suggesting that they are genetically related and have a common ancestor.

### Genetic Implications for Anticoagulant Proteins

The ability to synthesize anticoagulant substances is the most valuable feature for *H. manillensis*. We therefore examined the composition of anticoagulant proteins, focusing on those with two Pfam protein domains: Hirudin (Pfam entry: PF00713) and Antistasin (Pfam entry: PF02822), which are the functional fundamental inhibitors of thrombin. To our knowledge, almost all known anticoagulant proteins in leeches contain such domains, as in NCBI Identical Protein Groups, and their members include Hirudin (most famous natural thrombin inhibitor), antistasin (inhibitor of blood coagulation factor Xa), hirustasin (inhibitor of tissue kallikrein, trypsin, alpha-chymotrypsin, and granulocyte cathepsin G), bdellin or bdellastasin (inhibitor of trypsin, plasmin, and acrosin), and decorsin (inhibitor of platelet aggregation).

As in *H. manillensis*, 16 proteins contained either the Hirudin or Antistasin domain. According to their annotations, most of them resembled the commonly known anticoagulants in leeches including Hirudin (Swiss-prot ID: Q07558), Guamerin (Swiss-prot ID: P46443), and Bdellastasin (Swiss-prot ID: P82107). However, one gene of the Antistasin family (evm.model.Contig00032.80) was different from any known protein in the NR or Swiss-prot database; hence, we inferred that it is a novel protein potentially resulting from intense gene recombination and may have different functions. Further, to determine the presence of this gene encoding this protein, we determined its expression levels, and the average FPKM value was 11.92 among six samples.

**Figure 6:**
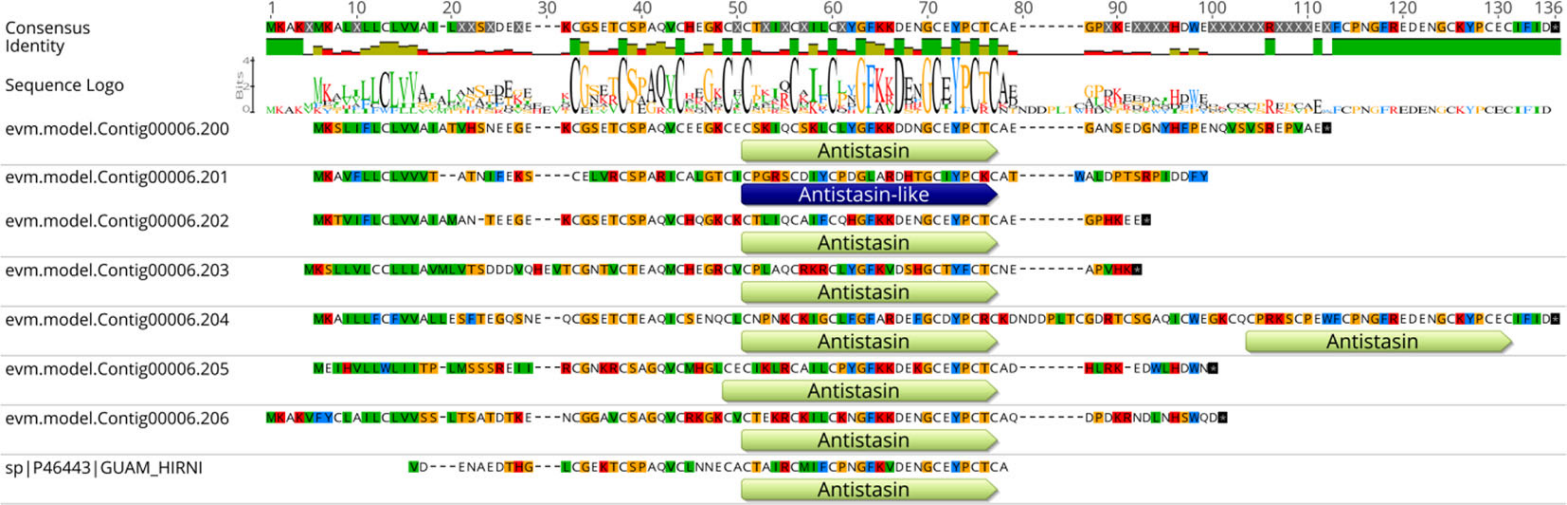
The distribution of Antistasin domains in the gene cluster on contig00006 of the assembly of *H. manillensis*. The gene IDs are shown on the left. The consensus sequence of the alignment is shown on the top of the figure. After the identity of the sequences and the sequence logo, the variations among these sequences are shown.

Moreover, while analyzing these anticoagulant proteins, we noticed an interesting pattern in a gene cluster on Contig 6. There were seven linearly distributed genes: from evm.model.Contig00006.200 to evm.model.Contig00006.206, having conserved sequences encoding Guamerin (except for evm.model.Contig00006.201) (**Figure 6**). Multiple gene duplications were expected for these genes, along with a greater requirement of Guamerin, an elastase-specific inhibitor.

## Conclusion

This study is the first to present the annotated genome sequence assembly of the Asian Buffalo leech. We generated a draft long-read genome assembly of 151.8 Gb and a N50 scaffold size of 2.28 Mb. The assembled genome was predicted to contain 21,005 protein-coding genes and up to 29.6 Mb (17.3%) of repetitive elements. In total, 17,865 genes were annotated. Genes encoding anticoagulants such as Hirudin were present and a gene cluster encoding Guamerin was identified. This annotated draft genome for *H. manillensis* provides valuable data for captive leech breeding and facilitates further studies on the phylogeny and biological features of *H. manillensis*, such as the biosynthesis of anticoagulant proteins and genetic adaptions for blood sucking (Abdelgabar and Bhowmick, 2003; Struck et al., 2007; Thompson, 2010; Struck et al., 2011).

## Data Availability Statement

Raw data have been deposited in NCBI SRA (sequence Read Archive) database with the project accession PRJNA475873 (genome) and PRJNA477611 (survey). The SRA accessions are SRR7415780 (genome) and SRR7415797 (survey), respectively. The RNA sequence dataset supporting the present results is available in the repository of NCBI Sequence Read Archive (SRA) with the following accession Number: SRX6035209 ~ SRX6035214.

## Ethics Statement

Mature *H. manillensis* samples were collected from ponds in Hechi City, Guangxi Province, China. As prescribed by “Law of People's Republic of China on the Protection of Wildlife” and “Regulations for the Implementation of the People's Republic of China on the Protection of terrestrial Wildlife” (State Council Decree [1992] No. 13), specimen collection did not require any ethical or institutional approval because *H. manillensis* is not endangered or protected by any current law. The care and treatment of animals in this study were in accordance with the Guideline for the Care and Use of Laboratory Animals in China.

## Author Contributions

D-LG, S-QX, and QQ designed the study. L-BM, Y-KL, and Z-ZW collected the samples. DM and YL conducted the sequencing analysis. D-LG and JY were involved in the data analyses. D-LG, JY, and S-QX wrote the manuscript. All authors read and approved the final manuscript.

## Funding

This study was financially supported by the Excellent Doctor Innovation Project of Shaanxi Normal University (S2015YB03), the Fundamental Research Funds for the Central Universities (GK201903063; GK201702010), the National Natural Science Foundation of China (No.31872273), and the Key Research and Development Program of Shaanxi Province (2018ZDXM-SF-079).

## Conflict of Interest

Authors YL and DM were employed at Nextomics Biosciences.

The remaining authors declare that the research was conducted in the absence of any commercial or financial relationships that could be construed as a potential conflict of interest.

## Abbreviations

BUSCO, Benchmarking Universal Single-Copy Orthologs; LTRs, terminal repeat retrotransposons; MITE, miniature inverted-repeat transposable elements; TE, transposable element; SSR, simple sequence repeat; GO, gene ontology; KEGG, Kyoto Encyclopedia of Genes and Genomes; TrEMBL, Translated EMBL Nucleotide Sequence Data Library.
