# Supplementary material for: Draft Genome of the Asian Buffalo Leech Hirudinaria manillensis
Source: Front Genet. 2020 Jan 16;10:1321. doi: 10.3389/fgene.2019.01321 (PMC6977106; doi:10.3389/fgene.2019.01321)
Supplement: Supplementary file 3 [file Table_1.docx]

Table S1: Statistics for genome sequencing of the Asian Buffalo leech.

| Library | Read  length (bp) | Total  data (Gb) | Sequence  coverage (×) | Read  number | Total  Data (Gb) | Sequence  coverage (×) | Read  number |
| --- | --- | --- | --- | --- | --- | --- | --- |
| Short read | 150 | 12.3 | 80.1 | 81,897,022 | 12.2 | 79.9 | 81,755,333 |
| Long read | 500~89445 | 15.1 | 98.3 | 1,597,978 | 14.9 | 97.7 | 1,505,497 |

Note: The coverage calculation was based on the K-mer estimated genome size of 153.1 Mb. Sequence coverage was the mean number of times a base was sequenced.
