# Supplementary material for: Draft Genome of the Asian Buffalo Leech Hirudinaria manillensis
Source: Front Genet. 2020 Jan 16;10:1321. doi: 10.3389/fgene.2019.01321 (PMC6977106; doi:10.3389/fgene.2019.01321)
Supplement: Supplementary file 4 [file Table_2.docx]

Table S2: Statistics for annotated repeats in the Asian Buffalo leech genome

| Type of repeat |  | Number | Length (bp) |
| --- | --- | --- | --- |
| DNA transposons: |  |  |  |
|  | CMC-EnSpm | 153 | 13045 |
|  | Crypton-H | 1849 | 285410 |
|  | IS | 54 | 56226 |
|  | Maverick | 3 | 3980 |
|  | Merlin | 75 | 31223 |
|  | MuLE-MuDR | 143 | 56421 |
|  | MuLE-NOF | 35 | 32151 |
|  | PiggyBac | 37 | 18118 |
|  | TcMar-Pogo | 230 | 71171 |
|  | TcMar-Tc1 | 705 | 456398 |
|  | TcMar-Tc2 | 62 | 13381 |
|  | Zator | 38 | 6984 |
|  | hAT-Ac | 151 | 91362 |
|  | hAT-Charlie | 330 | 69131 |
|  | hAT-Tip100 | 135 | 40297 |
|  | hAT-Tol2 | 22 | 7653 |
|  | hAT-hATm | 96 | 48377 |
| LINE: |  |  |  |
|  | CR1 | 550 | 464240 |
|  | CR1-Zenon | 27 | 15507 |
|  | Dong-R4 | 79 | 63927 |
|  | I | 26 | 8562 |
|  | I-Jockey | 589 | 102582 |
|  | L1 | 26 | 106916 |
|  | L2 | 2163 | 862289 |
|  | R2 | 436 | 1015491 |
|  | R2-Hero | 383 | 665984 |
|  | R2-NeSL | 17 | 49264 |
|  | Tad1 | 12 | 4692 |
| LTR: |  |  |  |
|  | Copia | 446 | 260523 |
|  | Gypsy | 5246 | 1066452 |
|  | Pao | 69 | 37355 |
| RC: |  |  |  |
|  | Helitron | 2628 | 596186 |
| Low_complexity |  | 16760 | 901055 |
| SINE? |  | 158 | 10721 |
| Satellite |  | 128 | 196570 |
| Simple_repeat |  | 272624 | 17796129 |
| Unknown |  | 15944 | 5397997 |
| snRNA |  | 21 | 29502 |

LINE, long interspersed nuclear repeats; LTR: long tendent repeats; RC: rolling circle; SINE, short interspersed nuclear elements.
